# Mr. Stafford on Lancetted Stilettes in Stricture

**Published:** 1831-01-01

**Authors:** 


### XXVIII.

### Lancetted Stilettes in Stricture.

Mr. Stafford has very laudably published an appendix to, instead of a new edition of his work on strictures, for the accommodation of those who hold the second edition. He informs us that he has now operated on more than forty cases of permanent stricture, of the worst description, without a single failure. "In no instance has there been a false passage made, nor has the cutting through the contracted part caused pain, hæmorrhage, inflammation, or any other unfavourable symptom." The hardened structure of the stricture has been absorbed, and no relapse has taken place, as far as he knows. In addition to these cases he has, on two different occasions, divided through an enlarged third lobe of the prostate gland, which in one case had caused total, and in the other, partial retention of urine. In both cases the patients recovered the complete power of the bladder. We shall introduce a single case out of 18 here published.

"*Case* 11. A distinguished general officer, rather more than sixty years of age, and of a shattered constitution, from hard service in India, the Peninsula, and other countries, placed himself under my care with a diseased urethra, which he had dreadfully suffered from for more than thirty years. The canal was irregularly thickened and contracted to four inches in extent, which began exactly four inches distant from the orifice, and terminated at the prostate gland. He had frequently undergone courses of bougies, and the application of caustic, without any beneficial result; at length ulceration took place in the urethra, the urine was extravasated, and a fistulous passage ensued in the perineum. In course of time this healed up, since which the passage gradually got worse, until it would not admit any thing larger than a No. 3 bougie, which, for the last six months, he has always been obliged to pass into the bladder before he could make water, and if he did not succeed in that operation he invariably suffered from retention of urine. His bladder, also, had partly lost its power, expelling only about half its contents. The urine was always extremely fetid and of a turgid dark colour, and there was very frequently deposited a considerable quantity of sediment at the bottom of the chamber-pot of a tenacious thick mucus, such as is secreted in diseases of the prostate gland.

Having given up all hope of deriving any benefit from the common mode of treatment, he made up his mind to have the diseased structure gradually divided by the stilette. This I accomplished by four different operations, from time to time, and during the whole treatment no unfavourable symptom occurred. At length I was enabled to introduce a No. 12 flexible catheter into the bladder, which I left there a few days. After this time I discharged my patient, allowing him to pass bougies for himself twice a week until the cure was completed. I have since heard from him by letter, in which he states—

'I can now decidedly say from experience that your operation has succeeded to my fullest expectation. I have never any difficulty in making water, and I have invariably passed the bougie you desired me (No. 13) without stoppage, pain, or difficulty, every fourth day, and I can even pass a large No. 14.' He can now expel the whole contents of his bladder: the urine is never fetid, and the prostatic secretion has entirely disappeared."

